# Neighborhood Social Processes and Adolescents' Depressive Symptoms: The Intervening Role of Neighborhood Self‐Efficacy

**DOI:** 10.1002/jcop.23180

**Published:** 2025-01-19

**Authors:** Kristen A. Berg

**Affiliations:** ^1^ Jack, Joseph and Morton Mandel School of Applied Social Sciences Case Western Reserve University Cleveland Ohio USA; ^2^ Center for Health Care Research and Policy, Population Health and Equity Research Institute Case Western Reserve University School of Medicine/MetroHealth Medical Center Cleveland Ohio USA

## Abstract

Via observational data from the Project on Human Development in Chicago Neighborhoods collected between 1994 and 2001, this study examined the degree to which neighborhood disorder, collective efficacy, and youth‐centered institutional resources are directly associated with adolescents' depressive symptoms across time, and the mediating role of adolescents'neighborhood self‐efficacy. Latent variable structural equation models were estimated among an unweighted representative sample of 1448 adolescents (59% male, mean age 15.19), across 79 neighborhoods in Chicago, to examine the direct effects of neighborhood disorder, collective efficacy, and availability of youth‐centered resources at baseline (measured at timepoint 1; reported by an independent sample of Chicago adults) on adolescents' depressive symptoms (measured a timepoint 3), and the mediating effect of adolescents' neighborhood‐anchored self‐efficacy (measured at timepoint 2). Indirect effects were assessed using bootstrap testing. Adolescents' neighborhood self‐efficacy partially mediated the effects of greater social and physical neighborhood disorder (indirect *β* = 0.03, 95% CI [0.008, 0.075]) and less availability of neighborhood youth‐centered resources (indirect *β* = −0.01, 95% CI [−0.030, −0.001]) on depressive symptoms over time. Adolescents who perceived themselves to have greater neighborhood self‐efficacy reported lower levels of depressive symptoms across time (*β* = −0.13, 95% CI [−0.24, −0.03]), as did those in neighborhoods with greater availability of youth‐centered resources (*β* = −0.11, 95% CI [−0.19, −0.03]). Those living in neighborhoods with more disorder reported lower neighborhood self‐efficacy (*β* = −0.24, 95% CI [−0.36, −0.11]). Neighborhood‐anchored self‐efficacy may be one mechanism by which adolescents internalize their neighborhood environments in ways that, over time, affect depressive symptoms. Interventions aimed at fostering community environments that nurture opportunities for youth to build efficaciousness may be promising for mitigating adolescent depression.

## 1 Introduction

Nearly 5 million American adolescents experienced at least one major depressive episode in 2022 and, of those, nearly 3.6 million reported consequent impairment in their family or peer relationships or academic functioning (National Institutes of Mental Health 2023). Adolescents experiencing depression experience poorer self‐esteem (Lee and Lee 2023), worse academic performance (Diaconu‐Gherasim and Măirean 2020; Moè 2015), and are at higher risk of engaging in substance use (Wilkinson, Halpern, and Herring 2016). Furthermore, young people experiencing depression are at higher risk of experiencing continued symptomatology into adulthood if untreated (Fergusson, Boden, and Horwood 2007; Patton et al. 2014). Compared to adults, depressive symptoms in adolescents are often undetected because of their consistency with some normative behaviors of adolescence such as irritability or tiredness (Thapar et al. 2012). Physiological and neurocognitive maturation during puberty catalyzes adolescents' heightened social and self‐awareness (Sebastian, Burnett, and Blakemore 2008), and concomitant changes in neural circuitry implicated in stress reactivity increase adolescents' vulnerability to developing depressive symptoms when faced with chronic environmental stressors (Andersen and Teicher 2008; Thapar et al. 2012).

Research in recent decades has increasingly considered the relevance of neighborhood environments to adolescents' health and, more specifically, how social processes within those neighborhoods may affect depressive symptoms (Dawson et al. 2019; Hurd, Stoddard, and Zimmerman 2013; King, Huang, and Dewan 2022; Nebbitt and Lombe 2009; Saleem, Busby, and Lambert 2018). Bioecological and social determinants perspectives of depression underscore the role of distal factors, such as neighborhood social environments, in addition to more proximal individual or family characteristics (Remes, Mendes, and Templeton 2021; Robins et al. 2024). Research suggests that adolescents who perceive their neighborhoods as more threatening given disorder or violence, more socioeconomically distressed, or less socially cohesive tend to report more depressive symptoms (e.g., Aneshensel and Sucoff 1996; Barr 2018; Snedker and Herting 2016). However, comparatively few studies have examined psychological mechanisms by which adolescents may internalize aspects of neighborhood in ways that manifest as depressive symptoms (Browning et al. 2013; Dupéré, Leventhal, and Vitaro 2012; Vilhjalmsdottir et al. 2016). One such mechanism may be neighborhood self‐efficacy (i.e., how capable youth feel of navigating their neighborhood and avoiding conflict). This study builds on prior research by examining the extent to which neighborhood social processes of disorder, collective efficacy, and availability of youth‐centered resources affect adolescents' depressive symptoms both directly and indirectly through their neighborhood‐anchored self‐efficacy.

### 1.1 Neighborhood Ecologies and Adolescent Depressive Symptoms

Research on depression in adolescence has traditionally emphasized biological and family risk factors (Hammen, Rudolph, and Abaied 2014; Nolen‐Hoeksema and Hilt 2008). For example, adolescents who are female, older, whose mothers have depressive histories, and those from more socioeconomically disadvantaged families have demonstrated more depressive symptoms (Hammen et al. 2003; Lu 2019). However, ecologically informed perspectives consider how chronic or acute adversities within broader social contexts like the neighborhood impact youth's depressive symptoms, and research identifies both genetically predisposing and environmental stress factors (Heim and Binder 2012; Toenders et al. 2022). While the role of neighborhood characteristics as specific environmental strains has been more studied in adult samples (Barnett et al. 2018; Galea et al. 2007; Mair, Roux, and Galea 2008; Tomita, Labys, and Burns 2015), comparatively less research has examined the developmental period of adolescence.

Studies examining neighborhood correlates of adolescent depressive symptoms have measured both structural factors (i.e., residents' aggregated sociodemographics such as socioeconomic position) and social processes (i.e., residents' social interactions or resources such as collective efficacy, conflict and violence; or institutional resources such as community centers or parks) (Barr 2018; Hurd, Stoddard, and Zimmerman 2013; Natsuaki et al. 2007; Pabayo et al. 2016; Sampson, Morenoff, and Gannon‐Rowley 2002; Wickrama, Merten, and Wickrama 2012). Developmental research has outlined key protective mechanisms of neighborhoods: namely, Jencks and Mayer (1990) posited five mechanisms hypothesized to affect youth behavior. For example, per a collective socialization model, neighborhood adults facilitate children's well‐being by acting as role models or maintaining collective efficacy to enact shared values such as maintaining a socially and physically ordered community (e.g., one with low crime, physical decay, or litter) (Jencks and Mayer 1990). Additionally, the institutional resource model assumes that quality neighborhood resources such as libraries or parks provide safe spaces and support interactions that nurture youth's physical and emotional health, and that the absence of such resources may increase youth's risk of engaging in risky behaviors (Jencks and Mayer 1990). Similarly, Galster's (2012) synthesis of 15 mechanisms emphasizes that social‐interactive mechanisms such as collective efficacy may confer benefit to youth by maintaining shared social values in the neighborhood such as safety. Environmental mechanisms such as chronic exposure to deteriorated infrastructure may affect children by imparting a sense of powerlessness, and geographical mechanisms may affect youth through inadequate public services such as schools; and institutional mechanisms may affect youth through their access to quality public or private institutions such as parks, behavioral health services, or recreation resources (Galster 2012).

From this body of work, neighborhood social processes of disorder, collective efficacy, and institutional resources are theorized to affect adolescents' depressive symptoms.

#### 1.1.1 Neighborhood Disorder

Aneshensel and Sucoff's (1996) seminal work suggests that when adolescents frequently encounter neighborhood disorder (i.e., visible signs of physical deterioration and social disruption, such as graffiti, crime, or public alcohol consumption, that may indicate the breakdown of social control; Gracia 2014), they are more likely to view their communities as threatening, consequently increasing their susceptibility to depressive thoughts. Research in the following decades has corroborated the robust association between neighborhood disorder and internalizing symptoms (Mair, Roux, and Galea 2008; White et al. 2021). Some work suggests that adolescent girls may be particularly vulnerable to the deleterious mental health effects of neighborhood social disorder, especially when encountering groups of unsupervised male youth in public spaces during which girls may experience heightened concerns about potential sexual victimization threats (Browning et al. 2013; Popkin, Leventhal, and Weismann 2010). Similar findings on disorder and depression have been replicated in both larger national (Ford and Rechel 2012) and regional samples (Snedker and Herting 2016). Elements of disorder, such as vacant housing, may signify a neighborhood's inefficacy to form stable social capital, thus diminishing community social support for adolescents and families, and may also signal a neighborhood's disinvestment by the greater community (Wilson 1996, in Snedker and Herting 2016). Such disinvestment may be perceptible to adolescents and internalized in ways that heighten emotional distress during adolescence itself, and also persist into early adulthood (Barr 2018).

Indeed, findings from qualitative research have identified vacant infrastructure as eliciting feelings of sadness and perceptions of abandonment by their greater communities (Teixeira 2015). Qualitative findings by Mmari et al. (2014) illustrate the ways in which adolescents in disadvantaged urban neighborhoods perceive and navigate their environments, delineating that signals of physical disorder such as vacant homes or buildings convey lack of safety. Such findings may clarify mechanisms undergirding links identified in other research between, for example, greater physical neighborhood disorder and higher odds of children's and adolescents' anxiety and depression (Butler et al. 2012).

#### 1.1.2 Collective Efficacy

A body of research has examined the extent to which collective efficacy (i.e., neighborhood residents' activation of their collective social ties to fulfill a common goal; Sampson 2003) predicts residents' depressive symptoms. Considering collective efficacy's primary components of social control and social cohesion, such research theorizes that more collectively efficacious communities are better able to regulate or control the behavior of community members, thus mitigating problem behaviors such as vandalism or public fighting that impart psychosocial strain on other residents (Cutrona, Wallace, and Wesner 2006). Research also theorizes that more socially cohesive neighborhoods are more socially supportive among residents, buffering the psychosocial strains of problem behaviors if they do occur and thus attenuating depressive symptoms (Ahern and Galea 2011; Cutrona, Wallace, and Wesner 2006; Kim 2008). Compared to research testing such theoretical links in adult samples (Ahern and Galea 2011; Roberts, van Lissa, and Helbich 2021), fewer studies have examined relation between collective efficacy and depressive symptoms in adolescent samples. Earlier work has found that young adolescents living in neighborhoods less collectively efficacious demonstrated higher levels of combined depressive and anxiety symptoms 3 years later (Xue et al. 2005). Other research suggests that living in less collectively efficacious neighborhoods earlier in life predicts more overall behavior problems during adolescence (Wang, Choi, and Shin 2020), and that a neighborhood's collective efficacy may enhance the protective effects of family cohesion and emotional support against adolescents' suicide attempts (Maimon, Browning, and Brooks‐Gunn 2010).

#### 1.1.3 Institutional Resources

A smaller cluster of studies has examined associations between adolescents' access to neighborhood institutional resources and their depressive symptoms, and this research suggests that engagement in even one community‐anchored social activity (e.g., volunteering, club participation) may be protective against major depressive disorder (Delfin et al. 2022). Even when adolescents live in neighborhoods with fewer youth‐centered institutional resources such as libraries or Boys and Girls Clubs, those who report often utilizing such assets indicate lower levels of depressive symptoms both contemporaneously and across time (Olson and Goddard 2015; Urban, Lewin‐Bizan, and Lerner 2010). This suggests that such resources may afford youth supportive social connection, mentoring relationships, and opportunities to build efficaciousness in any number of recreational activities, and that these experiences may protect against depressed mood (Mahoney, Schweder, and Stattin 2002).

### 1.2 Adolescent Depressive Symptoms and Self‐Efficacy in the Neighborhood

Jointly, this literature suggests that adolescents exposed to negative social processes in their neighborhoods such as less collective efficacy, more disorder, or fewer youth‐centered institutional resources tend to experience more depressive symptoms (Delfin et al. 2022; Snedker and Herting 2016; Wang, Choi, and Shin 2020), but less clear is how those social processes may be internalized and manifest as symptomatology. When considering adolescents' neighborhoods as contexts shaping varying access to supportive resources and also affording opportunities to build competence and mastery over the environment, one such cognitive mechanism may be self‐efficacy. Self‐efficacy reflects how capable one feels for carrying out behaviors necessary to influence a desired outcome, and has been examined as a factor impacting the development of depressive symptoms (Bandura 1994, 1997). Self‐efficacy tends to increase over time during adolescence, as youth become progressively more independent and agentic (Schunk and Meece 2006) and transition to spending more time outside of their family environments. Because self‐efficacy beliefs are critical determinants of motivation, cognition, and affect, they are considered core ingredients of psychological well‐being (Bandura 1994). For example, an adolescent's belief in their capability to maneuver through neighborhood space while avoiding conflict (i.e., neighborhood self‐efficacy) connects characteristics of their neighborhood context to their agency and psychological well‐being. Feeling more efficacious to manage an environment's (e.g., a neighborhood's) challenges may buffer against the potentially detrimental effect of those challenges and lower one's vulnerability to depression (Bandura 1994; Smith and Betz 2002).

Self‐efficacy is a dynamic and domain‐specific construct and thus suggested to be most powerfully measured in relation to a focal environment (e.g., the neighborhood) (Bandura 1986, 1997). However, compared to research examining self‐efficacy as a global construct linking environmental demands to depressive symptoms (Boardman and Robert 2000; Lopez and Crea 2021), less work has examined the link between neighborhood‐anchored self‐efficacy and depressive symptoms. Among adult samples, seminal work by Ross and colleagues (Ross and Mirowsky 2009; Ross, Mirowsky, and Pribesh 2001) has elucidated how self‐efficacy‐related concepts, such as perceived control or powerlessness, could link stressful neighborhood environments with psychological well‐being. Living in more disordered or unsafe communities may elicit greater perceived powerlessness which, in turn, predicts more depression (Booth, Ayers, and Marsiglia 2012; Ross and Mirowsky 2009). To the author's knowledge, one study has examined the intervening role of adolescents' neighborhood self‐efficacy on the link between neighborhood and internalizing behavior (Dupéré, Leventhal, and Vitaro 2012). Dupéré, Leventhal, and Vitaro (2012) measured an intervening effect of neighborhood‐anchored self‐efficacy such that adolescents living in neighborhoods with more collective efficacy reported more neighborhood self‐efficacy which, in turn, was linked to fewer combined anxious and depressive symptoms over time. This body of research across both adult and adolescent samples suggests that neighborhood environments may be important in shaping individuals' sense of efficaciousness and control, with implications for depressive symptomatology.

### 1.3 Current Study

**Figure 1:**
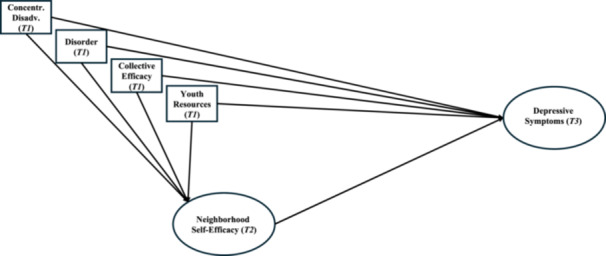
Conceptual model of the effects of neighborhood social processes (disorder, collective efficacy, youth‐centered resources) and adolescents' neighborhood self‐efficacy on adolescents' depressive symptoms.

Expanding upon prior research on adolescents' neighborhood environments and depressive symptoms, this study employed latent variable modeling to examine both direct and indirect effects of neighborhood disorder, collective efficacy, and the availability of youth‐centered institutional resources on adolescent depressive symptoms through neighborhood self‐efficacy (Figure 1). It was hypothesized that higher levels of disorder, lower levels of collective efficacy, and fewer youth‐centered institutional resources would be directly associated over time with more depressive symptoms and lower levels of neighborhood self‐efficacy among adolescents. It was further hypothesized that lower levels of neighborhood self‐efficacy would be linked with more depressive symptoms. Finally, less neighborhood self‐efficacy was hypothesized to partially mediate the effects of more disorder, less collective efficacy, and fewer institutional resources for youth on adolescents' greater depressive symptoms.

## 2 Methods

Data originated from the Project on Human Development in Chicago Neighborhoods (PHDCN). The PHDCN was a longitudinal study of over 6,000 children, begun in 1994, conducted to ascertain the role of individual‐, family‐, and neighborhood‐level characteristics on the developmental trajectories of Chicago youth (Earls and Visher 1997; Sampson 2012). The current study examined the effect of adolescents' neighborhood characteristics on their later depressive symptoms, both directly and indirectly through their neighborhood‐anchored self‐efficacy. This study was approved by the Case Western Reserve University Institutional Review Board.

### 2.1 Study Context

Full design and methodological detail on PHDCN has been published elsewhere (Earls and Visher 1997). In brief, the PHDCN includes two key components: first, the cross‐sectional Community Survey (PHDCN‐CS) measured sociodemographic patterns in all 342 Chicago neighborhoods (i.e., census tract clusters) from interviews with a representative probability sample of almost 9,000 Chicago adults from 1994 to 1995. Adults participating in the PHDCN‐CS responded to questions regarding their perception of neighborhood conditions such as social and physical disorder, social cohesion, and available social service resources. Stratified random probability sampling by racial‐ethnic composition and socioeconomic status generated a subsample of 80 neighborhoods which comprised the geographic units of analysis comprising a second key study component, the Longitudinal Cohort Study (PHDCN‐LCS).

The PHDCN‐LCS followed an independent sample of over 6,200 Chicago youth across 7 years at three different waves, the first of which (T1; 1994 through 1997) approximately aligned with data collection of the PHDCN‐CS. The second wave (T2) of data collection occurred from 1997 through 1999, and the third wave (T3) from 2000 through 2001. Seven cohorts of children were followed throughout all waves of the PHDCN‐LCS: those who, at baseline/T1, were 0, 3, 6, 9, 12, 15, and 18 years old. In this study, PHDCN‐LCS data at all timepoints are used for adolescent variables and PHDCN‐CS data from the 80 neighborhoods comprising the PHDCN‐LCS are used to assess neighborhood variables.

#### 2.1.1 Study Participants

The current study's analytic sample includes two cohorts of youth: those who were approximately 9 and 12 years old at T1 of the PHDCN (*N* = 1,448). At T1, 1,091 youth were screened as eligible for inclusion in cohort 9 of the PHDCN‐LCS. At a response rate of 75.9%, 828 LCS cohort 9 interviews were completed. Also at T1, 1,103 youth were screened as eligible for inclusion in cohort 12 of the PHDCN Longitudinal Cohort Study. At a response rate of 74.3%, 821 LCS cohort 12 interviews were completed. From a starting T1 sample of *N* = 1,649 (inclusive of both cohorts 9 and 12), only youth whose primary caregivers in the study were maternal figures (e.g., mother, adoptive mother) were included to maintain consistency in the study's maternal depression covariate, resulting in a reduced sample of 1,448.

Exploratory analyses were run to assess the sample's missingness on the outcome according to severity of depressive symptomatology at T1, across sex, ethnicity, and maternal caregiver depression, household income, and whether or not youth moved within or outside of Chicago between T1 and T3. Across all variables, participants were most likely to be missing data on at least one T3 depressive symptom item (36.6%), followed by scores on caregiver depression (25.6%). Exploratory analyses assessing missingness indicated no significant differences in mean depressive symptoms scores at T1 between youths who had data at T3 (*M* = 2.41, SD = 2.61) compared to those who did not (*M* = 2.39, SD = 2.58), *t*(1,425) = −0.09, *p* = 0.92, nor were differences detected at T2 between youths who had data at T3 (*M* = 3.45, SD = 3.08) compared to those who did not (*M* = 3.40, SD = 2.89), *t*(1,169) = −0.24, *p* = 0.81. Chi‐square tests of difference did not suggest association between missingness on the outcome (depressive symptoms T3) and sex (*χ* ^2^ (1) = 1.12, *p* = 0.29), ethnicity (χ^2^ (3) = 1.59, *p* = 0.66), nor caregiver depression (χ^2^ (1) = 1.87, *p* = 0.17). However, youth missing scores on depressive symptoms at T3 were in families with lower average per‐capita household income (*M* = 5.24, SD = 4.40) compared to those with complete scores (*M* = 5.98, SD = 5.02), *t*(1,357) = 2.54, *p* = 0.011. Chi‐square tests of difference suggested meaningful association between residential mobility after T1 and missingness on depressive symptoms at T3, but no mean differences in depressive symptoms at T1 were observed between youths who did not move during the length of the study, youths who moved but stayed within Chicago, and youths who moved and left Chicago, *F*(3, 1,423) = 0.89, *p* = 0.44. Similarly, no differences between residential mobility groups were observed in mean symptomatology at T2, suggesting that missingness on the outcome at T3 and residential mobility were linked through attrition from the study as a whole.

### 2.2 Measures

#### 2.2.1 Depressive Symptoms

Depressive symptomatology was operationalized by adolescents' self‐rated responses at T3 to nine items of the *DSM*‐oriented *Affective Problems* subscale within a shortened version of Achenbach and Rescorla's (2001) *Youth Self‐Report* (YSR). Items measured past 6‐month experience of symptoms such as changes in sleep patterns and energy levels (e.g., “underactive/lacks energy”), depressed mood (e.g., “unhappy, sad, or depressed”), eating patterns (e.g., “doesn't eat well”), or self‐blame (e.g., “feels too guilty”). All items were scored on a scale from 0 (“not true”) to 2 (“very or often true”), and higher scores indicate higher levels of depressive symptomatology. The subscale has been shown to discriminate between youth with dysthymic and major depressive disorders from those who do not (Ebesutani et al. 2010). In the current sample, youth who had been diagnosed with major depressive disorder with the *Diagnostic Interview Schedule for Children* (DISC 4; Costello et al. 1982) demonstrated significantly higher levels of depressive symptoms, from the *Affective Problems* subscale, compared to those without diagnosis (*F* [1, 822] = 14.57, *p* = 0.000) (Costello 1996). Per Achenbach and Rescorla (2001), raw scores are used in model estimation. The scale's internal consistency as measured by Cronbach's *α* is 0.70 in this sample. Depressive symptomatology was modeled as a latent factor.

#### 2.2.2 Neighborhood Self‐Efficacy

Neighborhood self‐efficacy was modeled as a latent factor measured by adolescents' responses at T2 to a 6‐item PHDCN‐created neighborhood self‐efficacy scale (Earls et al. 2007). Items were presented to youths in two stages of forced‐choice response. For example, participants were read two joint statements such as “Some kids feel like they can do things to go places within a few blocks of their home safely, BUT’ ‘Other kids feel that they can NOT be sure about getting places within a few blocks of their home safely” and then asked if their chosen statement was “sort of true” or “very true” about them. Scores were then combined and re‐coded into a final scale with values ranging from 1 (“very untrue”) to 4 (“very true”) such that higher scores on all items indicated higher levels of neighborhood self‐efficacy. The scale's internal consistency, as measured by Cronbach's *α*, is 0.58 in this sample. Similar levels have been reported by other studies using this measure (Sharkey 2006). Neighborhood self‐efficacy was specified as a latent construct to both model common variance among the measure's six items and account for the items' measurement error.

#### 2.2.3 Neighborhood Social Processes

Adolescents' aggregated neighborhood scores of disorder, collective efficacy, and available institutional resources for youth were modeled at T1. All variables originated from the PHDCN‐CS and were modeled as observed variables.

#### 2.2.4 Disorder

Neighborhood disorder combined two three‐item subscales measuring social and physical disorder. Social disorder assessed the degree to which PHDCN‐CS respondents perceived threatening social activities such as public drinking or open selling or use of drugs. Physical disorder measured symptoms of physical neighborhood deterioration such as presence of vacant or abandoned structures or litter. For both subscales, participants rated how problematic, on a scale from 1 (“not a problem”) to 3 (“big problem”), they perceived each symptom of disorder to be in their neighborhoods. Responses across both subscales were combined and averaged to create a possible neighborhood‐level score range of 1 to 3 with higher scores signifying greater disorder. The measure's reliability is 0.89 (Raudenbush and Sampson 1999).

#### 2.2.5 Collective Efficacy

Consistent with measurement in key PHDCN studies examining collective efficacy (Morenoff, Sampson, and Raudenbush 2001; Sampson, Raudenbush, and Earls 1997), this variable combined scores from two five‐item subscales measuring participants' shared expectation in their neighborhoods for social control (e.g., “Neighbors would break up a fight in front of your house where someone was being beaten or threatened”) and social cohesion (e.g., “People around here are willing to help their neighbors”). All items on the resulting summary measure are coded from 1 (“strongly disagree”) to 5 (“strongly agree”), with higher scores indicating more collective efficacy. Internal consistency for this measure is 0.80 (Browning et al. 2013; Sampson, Raudenbush, and Earls 1997).

#### 2.2.6 Youth‐Centered Resources

PHDCN‐CS respondents' perception of available youth‐centered neighborhood institutional resources was measured by dichotomous responses to six items inquiring about the presence of neighborhood services such as recreation programs, clinics, or mentoring programs such as Big Brothers/Big Sisters. Individual responses were standardized across all neighborhoods to a mean of 0 and standard deviation of 1, creating a continuous measure with scores ranging from −3.57 to 0.76.

#### 2.2.7 Confounding Variables

Informed by VanderWeele (2019) principles of confounder selection, analyses adjusted for adolescent age, sex, and racial and ethnic identity, maternal depression, per capita household income, and neighborhood concentrated disadvantage index scores. Age is measured continuously and sex dichotomously with female youth as the reference group. Two dummy variables were created to measure racial and ethnic identity, with Hispanic youth as the reference group. Maternal depression was measured dichotomously by the Composite International Diagnostic Interview short form (CIDI‐SF; Kessler and Mrozek 1997) inquiring whether, in the past 12 months, caregivers had experienced sadness, emptiness, or depression for 2 weeks or longer. Positive responses indicate depression, and negative responses (reference group) indicate no depression. Due to missing data on this variable at T1 (*N *= 127 valid cases), T2 measurement was used. Per capita household income represents maximum total income earned by each adolescent's household in the past tax year divided by household size. Residential mobility was measured categorically to delineate whether, throughout the duration of the study, youth remained in their original neighborhood of residence (reference group), moved to a different neighborhood within the City of Chicago, or moved to a neighborhood outside of Chicago. The PHDCN‐created neighborhood concentrated disadvantage index score incorporates 1990 census data including the percentage of families living below the poverty line, percentage of families receiving public assistance, percentage of female‐headed households, and percentage of unemployed residents. A deviation score, values on this measure range from negative to positive (Sampson and Bartusch 1998).

### 2.3 Data Analysis

All analyses were conducted using the Mplus version 8.1 (Muthén and Muthén 2017). Maximum likelihood estimation with robust standard errors was used for its robustness to non‐normality and variables' quasi‐interval nature as well as its robustness to nonindependence of observations with the sample's complex structure of adolescents nested within neighborhoods (Rabe‐Hesketh and Skrondal 2004). Examination of indicators' intra‐class correlation coefficients (ICCs) during early estimation of multilevel measurement modeling did not evidence sufficient difference in depressive symptoms between neighborhoods to justify specification of a full multilevel structural equation model. ICC values across depressive symptomatology indicators ranged from 0.012 to 0.061. Insufficient variance in depressive symptoms existed between neighborhoods for partitioning into both within‐cluster and between‐cluster covariance matrices. Missing data were handled by the full information maximum likelihood method employed during model estimation; though this approach handles missing data by drawing upon all possible information from available scores in the data, it assumes data to be missing at random.

#### 2.3.1 Structural Equation Model Specification

##### 2.3.1.1 Measurement Model

The core structural equation model was built in two steps. First, a correlated two‐factor confirmatory factor analysis (CFA) was estimated specifying the depressive symptoms and neighborhood self‐efficacy latent constructs and their reflective indicators. One factor loading from each latent factor to its marker item indicator was constrained to 1 to scale the factor and ensure identifiability. Error variances were specified for each reflective indicator, and regression coefficients of each error variance were fixed to 1.

##### 2.3.1.2 Structural Model

Second, a structural model was estimated to evaluate the degree to which neighborhood social process predictors of disorder, collective efficacy, and youth‐centered resources at T1 were associated with adolescents' depressive symptoms at T3, both directly and indirectly through neighborhood self‐efficacy at T2. The depressive symptoms outcome (T3) was regressed upon all control variables (T1). Given that self‐efficacy is expected to change (i.e., increase) over time as adolescents age, neighborhood self‐efficacy (T2) was regressed upon age as a covariate (T1). Additionally, given that household income is known to affect how adolescents' families select into specific neighborhood environments which thus affect neighborhood self‐efficacy, neighborhood self‐efficacy (T2) was also regressed upon household income as a covariate (T1). In addition to structural paths between observed neighborhood predictors, neighborhood self‐efficacy, and depressive symptoms, correlations were specified amongst neighborhood disorder, collective efficacy, availability of youth‐centered resources, and concentrated disadvantage.

##### 2.3.1.3 Testing Indirect Effects

To test the significance of indirect effects (Gunzler et al. 2013) we employed bias‐corrected bootstrapping with 10,000 resamples, where random samples are repeatedly drawn with replacement from the original data set and indirect effects are calculated in each resample. This process generates an empirical approximation of the sampling distribution of the indirect effect, permitting the construction of 95% confidence intervals. If generated confidence intervals did not contain zero, neighborhood self‐efficacy was interpreted to transmit some effect of the relevant neighborhood social process to adolescents' depressive symptoms as the indirect effect was interpreted to differ from zero at *p* < 0.05 (Hayes 2009).

##### 2.3.1.4 Structural Equation Model Fit

Strict reliance upon general rules and their cut‐off values for establishing model goodness of fit is broadly cautioned against in methodological literatures of structural equation modeling (see Kline 2016; Marsh, Hau, and Wen 2004). With such caution in mind, and with ultimate reference to conceptual underpinnings for interpretation of results, four indices were referenced to evaluate models' goodness‐of‐fit: Bentler Comparative Fit Index (CFI), Root Mean Square Error of Approximation (RMSEA), RMSEA confidence intervals, and standardized root‐mean‐square residual (RMR) (Byrne 2012; Hu and Bentler 1999; Kline 2016). The Chi‐square index was unsuitable for the current study due to the index's sensitivity to large sample sizes. Bentler CFI values of at least 0.90 and closer to 0.95 or above, RMSEA values less than 0.05 and closer to 0.0, RMSEA 90% confidence intervals with lower value near 0.0 but less than 0.05 and upper values less than 0.08, and standardized RMR (SRMR) values less than 0.05 were interpreted as suggesting better approximate fit between the covariance matrix implied by the study's SEM and the data's observed covariance matrix (Bentler 1990; Byrne 2012; Hu and Bentler 1999; Kline 2016). Modification indices were examined in considering post‐hoc respecification (Kline 2016), and models were respecified prioritizing conceptual and theoretical soundness.

Following similar approaches in observational research (e.g., Dawson et al. 2019) a stability analysis was performed in a subsample of adolescents excluding those with an earlier diagnosis of major depression disorder to assess the potential confounding role of severe depression. In total, 1,125 adolescents reported no major depression diagnosis, measured within an adapted version of the Major Depression Disorder module of the Diagnostic Interview Schedule for Children (Shaffer et al. 2000). All analyses were repeated in this reduced sample to ascertain the robustness of findings in a population less affected by severe symptomatology.

## 3 Results

Table 1 illustrates descriptive statistics for adolescent and neighborhood variables. Of the 1,448 youths included, the sample was predominately Hispanic (47.65%) followed by African American (34.74%) and White (14.02%) and included nearly equal numbers of male and female youth. On average, the sample was 10.61 years old (SD = 1.53) at T1, and 15.19 years old (SD = 1.57) at T3. Most adolescents (39.96%) lived in households earning between $10,000 and $29,000 in the previous year, and nearly a quarter reported earnings of less than $10,000. At T3, the sample reported 3.04 average symptoms of depression. Approximately 12% of the sample had moved out of Chicago by T3, 22% of youths moved by T3 but stayed within the city, and over a third of the sample had not moved by T3.

**Table 1:** Sample Demographics and Descriptive Statistics.

Variable	*M*(SD) *or n*(%)	Range^a^
Adolescent (*n *= 1,448)		
T1 Age	10.61 (1.53)	7.77–13.03
T3 Age	15.19 (1.57)	11.75–18.50
Male	727 (50.21)	
Racial/ethnic identity		
Hispanic	47.65 (690)	—
Black	34.74 (503)	—
White	14.02 (203)	—
Other or missing	3.59 (52)	
Baseline depression^b^	4.90 (58)	—
Maternal caregiver depression	37.46 (402)	—
Per capita household income	$5,769.77 ($4,857.52)	
Household income		
< $10 K	329 (23.77)	—
$10–$19,999 K	295 (21.32)	—
$20–$29,999 K	258 (18.64)	—
$30–$39,999 K	187 (13.51)	—
$40–$49,999 K	108 (7.80)	—
> $50 K	207 (14.96)	—
Residential mobility		
No move	548 (37.84)	
Move within Chicago	313 (21.61)	
Move outside of Chicago	179 (12.36)	
Missing	408 (28.18)	
T3 Depressive symptoms	3.04 (2.64)	0.00–15.00
T2 Neighborhood self‐efficacy	18.86 (3.43)	8.00–24.00
Neighborhood (*n* = 79)		
Disorder	1.87 (0.34)	1.19–2.44
Collective efficacy	7.19 (0.60)	6.09–8.89
Youth‐centered resources	−1.73 (0.88)	−3.57–0.76
Concentrated disadvantage	−0.43 (3.42)	−5.36–11.92

*Note:* T3 = wave/timepoint 3; T2 = wave/timepoint 2.

^a^ Range refers to observed scores.

^b^ Major depressive disorder diagnosis.

Initial model fit from the correlated two‐factor CFA measurement model demonstrated inadequate fit, with a CFI of 0.85, RMSEA of 0.050 with a 90% confidence interval (CI) [0.045, 0.056], and SRMR of 0.046. Post‐hoc examination of modification indices and consultation with literature examining clustered symptoms of depression encouraged four pairs of correlated measurement errors (i.e., item residuals) among depressive symptoms items. Two pairs linked items pertaining to adolescents' tiredness and low energy: “I do not have much energy” with “I feel overtired” (*r* = 0.25), and “I sleep less than most kids do” with “I feel overtired” (*r* = 0.18). Two other pairs linked items pertaining to adolescents' self‐blame and fatigue: “I feel too guilty” with “I feel worthless or inferior” (*r* = 0.26), and “I feel overtired” and “I feel too guilty” (*r* = 0.13). Three pairs of item residuals among neighborhood self‐efficacy items were correlated for double‐negative wording: “I cannot avoid fights in the neighborhood” with “I cannot avoid gangs” (*r* = 0.25), “I cannot avoid being scared on the way to school” with “I cannot avoid gangs” (*r* = 0.22), and “I cannot avoid fights in the neighborhood” with “I cannot avoid being scared on the way to school” (*r* = 0.15). One additional measurement error correlation was specified for items' content of conceptually opposite concepts: “I feel safe alone in my neighborhood” and “I cannot avoid being scared on the way to school” (*r* = 0.17). The revised measurement model demonstrated better fit, with a CFI of 0.97, RMSEA of 0.024 with 90% CI [.017, 0.030], and SRMR of 0.030.

**Figure 2:**
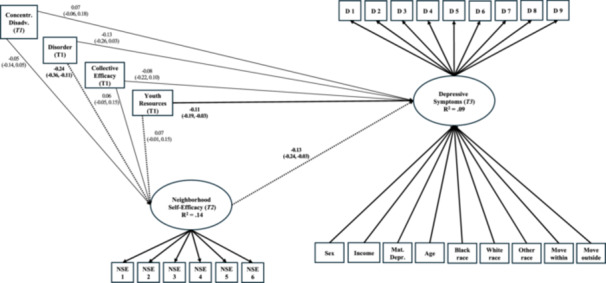
Model with standardized effects of associations between neighborhood disorder, collective efficacy, youth‐centered resources, and adolescents' neighborhood self‐efficacy on depressive symptoms. For visual clarity, residual covariances and factor loadings are not illustrated, nor are correlations among neighborhood disorder, collective efficacy, youth resources, and concentrated disadvantage, nor is the regression of neighborhood self‐efficacy on control variables age and household income. Dotted lines denote significant indirect effects through neighborhood self‐efficacy. Significant direct effects are shown by bolded betas and bootstrapped 95% confidence intervals.

After neighborhood predictors and covariates were integrated to the revised measurement model and structural paths added, model fit indices suggested an adequately fitting model with CFI of 0.93, RMSEA of 0.022 with CI [.019, 0.026], and SRMR 0.032. Older adolescents demonstrated significantly higher levels of depressive symptoms, as did female youth and those whose maternal caregivers reported depression. Figure 2 displays the model's structure of relationships among observed and latent variables and presents standardized estimates of path coefficients. Table 2 presents the model's standardized direct and indirect effects. Of the neighborhood social processes tested, only the availability of youth‐centered resources directly predicted depressive symptoms; specifically, adolescents living in neighborhoods with greater availability of youth‐centered resources reported lower levels of depressive symptoms across time (*β* = −0.11, 95% CI [−0.19, −0.03]), and those in less disordered neighborhoods reported greater neighborhood self‐efficacy (*β* = −0.24, 95% CI [−0.36, −0.11]). Adolescents who perceived themselves to have greater self‐efficacy in navigating their neighborhoods reported lower levels of depressive symptoms across time (*β* = −0.13, 95% CI [−0.24, −0.03]). The model accounted for 9% of the variance in adolescents' depressive symptoms and 13.9% of the variance in their neighborhood self‐efficacy.

**Table 2:** Standardized Direct and Indirect Effects and 95% Confidence Intervals of Neighborhood Social Processes on Adolescents' Neighborhood Self‐Efficacy and Depressive Symptoms.

Direct paths	Coefficient (95% CI)
Disorder and depressive symptoms	−0.13 (−0.255, 0.030)
Collective efficacy and depressive symptoms	−0.08 (−0.218, 0.096)
Youth resources and depressive symptoms	−0.11 (−0.189, −0.027)
Disorder and neighborhood self‐efficacy	−0.24 (−0.360, −0.114)
Collective efficacy and neighborhood self‐efficacy	0.06 (−0.049, 0.150)
Youth resources and neighborhood self‐efficacy	0.07 (−0.005, 0.147)
Neighborhood self‐efficacy and depressive symptoms	−0.13 (−0.240, −0.031)
Indirect paths	Coefficient (95% CI)
Disorder → neighborhood self‐efficacy → depressive symptoms	0.032 (0.008, 0.075)
Collective efficacy → neighborhood self‐efficacy → depressive symptoms	−0.008 (−0.029, 0.003)
Youth resources → neighborhood self‐efficacy → depressive symptoms	−0.010 (−0.030, −0.001)
Control paths	Coefficient (95% CI)
Sex at birth^a^ and depressive symptoms	−0.17 (−0.239, −0.087)
Per capita family income and depressive symptoms	−0.01 (−0.096, 0.074)
Maternal depression and depressive symptoms	0.09 (0.008, 0.165)
Age and depressive symptoms	0.08 (0.005, 0.162)
Black racial identity^b^ and depressive symptoms	−0.09 (−0.186, 0.008)
White racial identity^b^ and depressive symptoms	0.02 (−0.073, 0.116)
Other or missing racial identity^b^ and depressive symptoms	−0.01 (−0.075, 0.068)
Residential mobility^c^ within Chicago and depressive symptoms	0.00 (−0.082, 0.080)
Residential mobility^c^ outside Chicago and depressive symptoms	0.09 (−0.013, 0.214)
Age and neighborhood self‐efficacy	0.17 (0.099, 0.236)

^a^ Reference group includes female youth.

^b^ Reference group includes Hispanic youth.

^c^ Reference group includes youth who did not move during study period.

Finally, we examined the degree to which adolescents' neighborhood‐anchored self‐efficacy mediated any effect of neighborhood social processes on their depressive symptoms. Neighborhood self‐efficacy conveyed some effect of disorder (*β* = 0.03, 95% CI [0.008, 0.075]) and availability on youth‐centered resources (*β* = −0.01, 95% CI [−0.030, −0.001]) on depressive symptoms. Adolescents living in more socially and physically disordered neighborhoods, and those with fewer available youth‐centered resources such as recreation or health facilities, were measured to exhibit more depressive symptoms later in adolescence through the deleterious impact of such neighborhood characteristics on their feeling less efficacious in navigating their neighborhoods safely and without conflict.

Results from stability analyses when restricting only to adolescents with no earlier diagnosis of major depressive disorder generally support these primary findings, though with attenuated estimates, supporting the overall robustness of results. Youth living in neighborhoods with greater availability of youth‐centered resources reported fewer depressive symptoms across time (*β* = −0.09, 95% CI [−0.18, −0.01]), and those in less disordered neighborhoods reported greater neighborhood self‐efficacy (*β* = −0.20, 95% CI [−0.32, −0.07]). Greater neighborhood self‐efficacy predicted fewer depressive symptoms over time (*β* = −0.15, 95% CI [−0.26, −0.04]), and neighborhood self‐efficacy conveyed a similar effect of greater disorder on later depressive symptoms (*β* = 0.03, 95% CI [0.007, 0.069]).

## 4 Discussion

Informed by mechanisms models of endogenous neighborhood social processes and social cognitive theory, this study identified that adolescents living in more disordered neighborhoods reported lower levels of neighborhood‐anchored self‐efficacy which, in turn, was associated with greater depressive symptomatology. Findings also illuminated that adolescents living in neighborhoods with more youth‐centered resources reported fewer depressive symptoms over time and that neighborhood self‐efficacy may marginally convey some of that effect. This investigation builds upon existing research by examining not just which distal community factors may be pertinent to adolescents' depressive symptoms, but assessing neighborhood self‐efficacy as a specific cognitive mechanism by which such distal strains may be internalized by youth in ways that matter to depressive symptomatology.

Broadly, study findings comport with those of even the earliest empirical and conceptual research regarding the relevance of place to psychological well‐being (Faris and Dunham 1939). Findings that adolescents' feelings of neighborhood‐anchored efficaciousness may act as a psychological mechanism by which chaos in the broader community partially affect their depressive symptoms corroborate Ross and Mirowsky's (Ross and Mirowsky 2009) condition‐cognition‐emotion hypothesis. Living in more chaotic neighborhood spaces––for example, environments more disordered and less socially cohesive––can engender feelings of powerlessness and thus psychological distress if individuals are consistently presented with neighborhood situations over which they perceive little control (Geis and Ross 1998; Ross, Mirowsky, and Pribesh 2001; Ross and Mirowsky 2009). It follows, then, per self‐efficacy models of depression, that perceiving less efficaciousness to safely navigate chaotic neighborhood spaces may transmit effects of such environmental strain on feelings of depression.

Findings that adolescents living in communities with fewer institutional resources centered on youth well‐being reported more depressive symptoms over time are consistent with those of a smaller body of literature drawing upon frameworks of positive youth development to examine similar links between adolescents' use and availability of youth‐centered neighborhood institutions (e.g., recreation centers) and their depressive symptoms (Olson and Goddard 2015). Youth who engage with and access such resources may benefit from safe, supportive opportunities for adolescents to build interpersonal skills, develop warm relationships with respected mentors, practice conflict management and leadership skills, strengthen their community engagement and attachment, or safely access behavioral health services and counseling. The current study could not inventory individual types of institutional resources and their associations to depressive symptoms, but future research may seek to disentangle such effects. For example, it could be that specifically engaging with behavioral health treatment available in youths' neighborhoods alleviates depressive symptomatology. Further research may be informative for assessing which specific types of community‐anchored institutional investment most effectively and efficiently promote behavioral health.

Contrary to hypotheses, neighborhood collective efficacy at baseline was not significantly associated over time with adolescents' depressive symptoms nor their neighborhood self‐efficacy. This result was unexpected given the shared conceptual roots between community‐level (i.e., collective) efficacy and individual‐level neighborhood self‐efficacy as well as results from previous research suggesting positive association between the two (e.g., (Sharkey 2006). It may be that adolescents' individual efficacies may benefit from a community's enveloping collective efficacy at a certain threshold of collectivism, that is, perceptible to youth. In other words, a moderating effect may occur such that only in neighborhoods of more collectivist culture and social togetherness do youth's sense of agency, and thus behavioral health, benefit from witnessing neighborhood adults work jointly toward common goals for the neighborhood's greater good.

### 4.1 Strengths and Limitations

The current study has multiple strengths. First, while previous research has examined the role of stressful neighborhood conditions on young people's mental health, comparatively fewer studies have examined depressive symptoms extricated from combined anxious and depressive symptoms. Different diagnoses may imply differential mechanisms of effect and thus implicate different types of community‐relevant interventions for bolstering youth mental health. Second, the study capitalized on the PHDCN's measurement of self‐efficacy as a domain‐specific construct specifically anchored to youths' neighborhood environments, contributing evidence that young people's self‐efficacy is malleable construct rather than fixed or trait‐like (Schunk and Meece 2006). Methodologically, multiple sources of measurement reporting were capitalized upon by nature of the PHDCN‐CS's and ‐LCS's independent samples, mitigating possibility of adolescents reporting bleaker perceptions of their neighborhoods due to depressive cognitions and thus confounding their neighborhood evaluations. Additionally, the modeling of key family variables helps address concerns of selection bias; some household or family characteristics (e.g., household income, caregiver depression) that are theorized to affect adolescents' depressive symptoms may also affect their neighborhood of residence (Bergström and Ham 2010). Finally, the study exploited the longitudinal nature of data collection to test hypotheses of temporal association that neighborhood environmental features in earlier adolescence shape neighborhood self‐efficacy in mid‐adolescence, in turn shaping depressive symptoms in later adolescence.

Despite study strengths, findings should be considered in the context of limitations. Although the study's latent variable modeling approach accounted for measurement error present among the neighborhood self‐efficacy measure's items, the items' less‐than‐adequate internal consistency suggest that they likely do not fully capture a cohesive construct of neighborhood self‐efficacy. Future research might revise or expand this measure to build a more reliable assessment of adolescents' neighborhood self‐efficacy to better examine its relevance to youth mental health and community dynamics. Additionally, though the study measured neighborhood self‐efficacy at the second timepoint and depressive symptoms at the third timepoint, it may be relevant to consider a reverse‐order specification. Future research may test an alternative model examining how adolescents' depressive cognitions at an earlier timepoint may affect their sense of efficacy in the neighborhood at later timepoints and, reciprocally, how their earlier neighborhood self‐efficacy may affect their later depression as well as the stability of their self‐efficacy over time. Further, neighborhood measurements were only available for the first time point of study, and not all youth remained in their original neighborhoods throughout the course of the study. This methodological limitation precludes a more nuanced and dynamic measurement of neighborhood environments as affecting adolescents' outcomes. Relatedly, due to confidentiality restrictions limiting the ability to fully capture changes in participants' residences, time spent and change in neighborhood could not be modeled as a time‐varying covariate. Without such a time‐varying covariate, it could not be examined how youths' depressive symptoms and neighborhood self‐efficacies may have changed in tandem with the dynamic processes that unfold both within and across neighborhood environments.

Finally, although PHDCN data are now over two decades old, this study's findings potentially substantiate that the fundamental psychological processes linking neighborhood conditions to adolescent mental health are likely relatively stable over time. In the decades following the PHDCN study, for example, Mmari et al. (2014) demonstrated that adolescents across diverse global settings perceive their physical environment, including aspects of disorder, as affecting their health and well‐being. In the context of the current study, these consonant findings across different time periods and cultural contexts suggests a degree of universality to these relationships. Similarly, Barr (2018) measured neighborhood disorder to associate with more depressive symptoms in adolescence, with effects persisting into adulthood. This longitudinal perspective reinforces enduring neighborhood contexts of mental health, suggesting that the psychological mechanisms through which neighborhood conditions affect adolescents remain relevant and warrant ongoing research. Moreover, the concept of self‐efficacy as a mediator between environmental conditions and individual outcomes is grounded in well‐established psychological theories which have shown stability over time (Bandura 1977; Schunk and DiBenedetto 2021). Though specific neighborhood conditions may change over time (e.g., Berg et al. 2021), the processes by which adolescents interpret and respond to their environment through self‐efficacy likely remain consistent.

Recently published work on the decline of youth's independent activity, and the associated impacts on mental well‐being, additionally offer a compelling framework for considering why and how neighborhood‐anchored self‐efficacy may persistently convey at least some effects of the neighborhood on depressive symptoms. Gray, Lancy and Bjorklund (2023) posit that children's and adolescents' decreased opportunities for unsupervised, self‐directed activities in their neighborhoods have contributed to a decline in their sense of control over their lives, problem‐solving abilities, and emotional resilience. This notion aligns with our findings on the mediating role of neighborhood‐anchored self‐efficacy wherein adolescents who felt more self‐efficacious in navigating their neighborhoods later reported fewer depressive symptoms. Though neighborhood environments may shift over time, the fundamental psychological mechanisms linking environmental autonomy to mental health outcomes appear to remain salient. The PHDCN data's historical timing provides a unique analytical advantage, capturing adolescent‐environment interactions during a period of potentially greater neighborhood engagement. These data therefore may capture a crucial aspect of adolescent development that remains relevant in understanding contemporary youth mental health challenges.

### 4.2 Future Research and Implications

Emphasis for future research lies in study findings that physical aspects of the neighborhood environment, in addition to the oft‐studied social components, affect young residents' depressive symptoms. Rather than intervening upon social characteristics of a neighborhood (e.g., attempting to bolster residents' social cohesion), a community's built environment may be more actionable for promoting resilience from depressive symptoms. Indeed, experimental research has suggested that transforming vacant urban land into usable green space can lower symptoms of depression for neighborhood residents (South et al. 2018), and growing work documents links between exposure to green space and mental health benefits (Astell‐Burt and Feng 2019; Gonzales‐Inca et al. 2022). Additional research is warranted to examine these effects among younger residents.

Future research is also warranted for examining how adolescents' neighborhood self‐efficacy changes over time, and what actionable environmental factors strengthen it. In addition to structural changes in the neighborhood (e.g., revitalizing vacant parcels), attention to adolescents' social interactions within their neighborhoods may clarify specific avenues by which self‐efficacy can be strengthened. Bandura (1997) identifies mastery experiences and vicarious learning as two primary mechanisms by which self‐efficacy is nurtured, emphasizing that self‐efficacies are dynamic factors amenable to intervention. Adolescents who can safely practice conflict navigation with peers (e.g., during school, during community recreation programming) are more likely to achieve success and thus build feelings of mastery, increasing their confidence to carry out such behaviors in their neighborhoods. Similarly, a youth being able to observe how a respected peer or role model “take(s) care of themselves” (item language from the study's neighborhood self‐efficacy measure) in the neighborhood may serve as a vicarious experience fostering that youth's beliefs that they, too, are capable of exercising such behavior.

Future inquiry may also consider how the efficaciousness that young people build in certain life domains may affect their capacity for efficacy in other domains such as the neighborhood. Bandura (1997) specifies that while young people's self‐efficacies are best measured and conceptualized as domain‐specific beliefs, they do not operate in total isolation and thus may exert influence cross‐contextually. For example, youth who feel more capable of effecting control over relationships and interactions with family members (e.g., caregivers, siblings) to achieve positive outcomes (e.g., warmth, conflict resolution) may be better able to apply those learned skills during interactions in their neighborhoods to manage their safety and well‐being. Additional research is needed to clarify such dynamics.

Finally, study findings contribute to growing focus in research and policy on the social and physical circumstances that matter to young people's healthy development (Jansson et al. 2022; Villanueva et al. 2016). Community‐anchored initiatives aimed at improving youth behavioral health should prioritize providing adolescents tangible opportunities for positive engagement with their neighborhoods, fostering opportunities for mastery experiences and vicarious successes that build self‐efficacy. For example, youth presented with repeated opportunities to practice navigating peer conflict within the safe confines of supervised recreation center programming may build more efficacy in avoiding other interpersonal conflict. When youth feel more capable of handling such conflict, they may experience a higher threshold against feeling threatened, mitigating depressive symptoms and ultimately promoting resilience against behavioral health problems in the face of environmental strain.

## 5 Conclusion

Study findings suggest that self‐efficacy may be one cognitive mechanism by which aspects of the social and physical neighborhood environment are internalized in ways that affect adolescents' depressive symptoms. Findings also suggest that living in neighborhoods with more youth‐oriented institutional resources may offer protective benefits against adolescents' depressive symptoms. By prioritizing opportunities for positive engagement within neighborhoods and fostering efficacy‐building experiences, community initiatives can promote resilience and mitigate the impact of environmental strain on adolescent behavioral health. Interventions aimed at bolstering youth‐oriented neighborhood resources and supportive social networks may enhance adolescents' sense of competence and control over their environments, potentially mitigating the development of depressive symptoms.

## Conflicts of Interest

The author declares no conflicts of interest.

## Supporting information

Supporting information.

## Data Availability

Data originate from the Project on Human Development in Chicago Neighborhoods (PHDCN). Applications for individual use of PHDCN data are managed by the National Archive of Criminal Justice Data.
