# Supplementary material for: Neighborhood Social Processes and Adolescents' Depressive Symptoms: The Intervening Role of Neighborhood Self‐Efficacy
Source: J Community Psychol. 2025 Jan 19;53(1):e23180. doi: 10.1002/jcop.23180 (PMC11744059; doi:10.1002/jcop.23180)
Supplement: Supplementary file 1 — Supporting information. [file JCOP-53-0-s001.pdf]

Appendix

Pearson Correlations of Adolescent and Neighborhood Characteristics and Confounding Variables

		1	2	3	4	5	6	7	8	9	10	11	12	13	14	15	16	17	18	19	20	21	22	23	24	25	26	27	28
1	T3 DEP cry	–																											
2	T3 DEP eat	.184**	–																										
3	T3 DEP worthless	.269**	.239**	–																									
4	T3 DEP alone	.190**	.149**	.357**	–																								
5	T3 DEP guilty	.144**	.183**	.528**	.227**	–																							
6	T3 DEP overtired	.204**	.246**	.293**	.188**	.323**	–																						
7	T3 DEP sleep	.106**	.248**	.179**	.104**	.176**	.308**	–																					
8	T3 DEP energy	.181**	.226**	.262**	.197**	.105**	.388**	.180**	–																				
9	T3 DEP sad	.358**	.189**	.405**	.240**	.265**	.269**	.240**	.243**	–																			
10	T2 SE things w/ friends	-0.026	-0.006	-0.049	-.080*	-0.047	0.003	0.001	-0.040	-0.022	–																		
11	T2 SE gangs	-.090**	-0.035	-.067*	-0.017	-0.039	-0.040	-0.020	-.136**	-0.035	.145**	–																	
12	T2 SE scared	0.017	-0.057	-.085**	-0.028	-.064*	-0.026	-.088**	-.086*	-0.013	.157**	.294**	–																
13	T2 SE safe alone	-0.057	0.013	-.077*	-0.010	-.071*	-0.026	0.011	-0.024	-0.023	.236**	.114**	.266**	–															
14	T2 SE safe blocks	-.069*	-0.005	-0.054	-0.055	-0.050	0.004	0.033	-0.058	-0.053	.299**	.184**	.167**	.434**	–														
15	T2 SE avoid fights	-0.015	-0.033	-0.052	0.002	-0.039	0.013	-0.056	-0.026	0.007	.080**	.262**	.185**	.057*	0.045	–													
16	Disorder	0.004	-0.015	-0.010	0.041	-0.016	-.073*	-0.045	0.045	0.049	-.164**	-.107**	-.084**	-.183**	-.240**	-.119**	–												
17	Collective Efficacy	0.002	0.017	-0.050	-0.059	-0.043	0.036	0.028	-0.048	-.069*	.151**	.123**	.090**	.141**	.200**	.063*	-.718**	–											
18	Youth services	-.084*	-0.043	-.124**	-0.027	-.104**	-0.052	-0.060	0.061	-0.036	.061*	0.026	-0.004	0.056	.094**	-0.007	-.056*	.201**	–										
19	Age	0.025	.085*	0.003	0.048	0.042	.115**	.127**	.091**	0.032	-0.001	.064*	0.020	.129**	.137**	0.020	-0.004	0.012	-0.013	–									
20	Sex	-.325**	-.108**	-.086**	-.077*	-0.044	-.087**	-0.001	-.137**	-.150**	0.000	0.019	-0.007	0.025	0.020	-0.044	0.025	-0.027	0.018	-0.032	–								
21	Black ethnicity	0.021	0.019	-0.061	-0.013	-0.044	-.065*	-.108**	0.018	-0.053	0.016	-0.009	-0.013	-0.002	-0.001	-0.047	.102**	-.068**	0.051	0.015	-0.028	–							
22	White ethnicity	-0.056	0.013	0.001	-0.041	0.005	.069*	0.055	-0.056	0.008	.156**	.120**	.070*	.142**	.170**	.077**	-.378**	.339**	.201**	0.000	0.000	-.295**	–						
23	Other ethnicity	-0.030	0.050	-0.026	-0.012	-0.021	.067*	-0.011	0.041	-0.023	.058*	0.050	0.051	0.047	0.033	0.017	-0.037	0.014	0.036	-0.016	0.029	-.141**	-.078**	–					
24	Maternal depression	.075*	0.070	0.031	0.047	0.054	0.025	.071*	.078*	.073*	-0.034	0.009	-0.044	-.063*	-0.027	-0.040	.113**	-.085**	-0.021	.101**	0.001	0.005	-.077*	-0.001	–				
25	Per capita income	-0.044	0.000	-0.020	-.065*	-0.047	0.051	-0.026	-0.017	-0.021	.162**	.134**	.106**	.133**	.206**	.138**	-.424**	.347**	.126**	0.015	0.018	-.059*	.352**	0.017	-.111**	–			
26	Move within Chicago	0.003	-0.036	-0.040	0.044	-0.009	0.014	-0.019	.082*	0.035	-.068*	-0.023	-0.006	-0.027	-.082*	-.090**	.177**	-.192**	-0.037	0.025	-0.010	.096**	-.183**	-0.010	.131**	-.220**	–		
27	Move outside Chicago	-0.006	0.042	.069*	0.020	.066*	0.062	0.024	-0.009	0.004	0.058	-0.020	0.024	0.040	.075*	0.028	-.080*	0.003	-0.002	-0.051	0.023	-.067*	.121**	0.025	-0.019	.068*	-.299**	–	
28	Concentrated disadvantage	0.032	0.007	-0.036	0.041	-0.033	-0.057	-.074*	.067*	0.008	-.106**	-.083**	-0.050	-.129**	-.169**	-.115**	.607**	-.430**	0.011	0.007	0.015	.569**	-.375**	0.022	.109**	-.310**	.123**	-.104**	–

Note. ** Correlation is significant at the 0.01 level (2-tailed).
* Correlation is significant at the 0.05 level (2-tailed).
